# Operation of cognitive memory inhibition in adults with Down syndrome: Effects of maintenance load and material

**DOI:** 10.1371/journal.pone.0225009

**Published:** 2019-11-14

**Authors:** Elena Palomino, José María López-Frutos, María Sotillo

**Affiliations:** Department of Basic Psychology, Faculty of Psychology, Autonomous University of Madrid, Madrid, Spain; Radboud University, NETHERLANDS

## Abstract

**Background:**

Cognitive inhibition is one of the executive functions; this process over memory plays a fundamental role in recalling relevant information. The aims of this study were to understand the effects of maintenance load and stimuli on the operation of cognitive inhibition over memory in working memory tasks in adults with Down syndrome.

**Method:**

The study included 36 individuals with Down syndrome (mean age = 33.44 years, standard deviation = 7.54 years, 50% women) and 36 individuals with neurotypical development (mean age = 33.55 years, standard deviation = 7.52 years, 50% women). The participants performed a working memory task in which they had to solve an interference problem during the maintenance phase.

**Results:**

The Down syndrome group performed worse on cognitive inhibition over memory than the neurotypical development group. Both groups had lower recall with interference and under high-load conditions. In the neurotypical development group, memory was similar with both materials. The Down syndrome group performed better with non-social stimuli than with social stimuli.

**Conclusions:**

Understanding the variables that influence cognitive inhibition over memory will help in planning effective interventions for people with Down syndrome. Considering the results, special importance should be placed on work with social stimuli, at least in individuals with Down syndrome.

## Introduction

Working memory (WM) refers to a set of processes allowing mental representations to be temporarily accessible while performing cognitive and motor tasks [1–3]. Different proposals on WM have indicated its nature of temporary storage and its ability to keep information active while performing cognitive tasks. The ability to maintain the information available for processing varies depending on age, mood, the possibility of review, the difficulty of concurrent tasks, the maintenance load, and the presence of interference [4, 5].

Two of the issues that have attracted the greatest interest in conceptual proposals regarding the properties of WM are the amount of simultaneously active relevant information and how irrelevant information entry affects its maintenance. The first issue is related to the amount of relevant information that can be simultaneously actively maintained by an adult with neurotypical development (ND), which has been debated for years. In the 1950s, the amount was determined to be 7 ± 2 units of information [6]. However, current research indicates that the capacity for active information maintenance is 4 ± 1 information units [7]. In populations with intellectual disabilities (IDs), specifically in people with Down syndrome (DS), research suggests that the components of WM are not affected equally. Deficient performance has been found in verbal WM tasks that involve the active maintenance of information and therefore involve the articulatory loop [8]. In contrast, the performance of people with DS in spatial tasks, which involve the visuospatial sketchpad, presents a pattern similar to that of people with ND [9]. The second issue is that the WM can be affected by information that is not relevant to the current task, the processing of which should be inhibited. Cognitive inhibition generally intervenes in the disruption and ordering of cognitive processes. The ability to resolve the interference caused by irrelevant information to the task has been related to executive inhibitory mechanisms [10, 11]. Inhibition is considered a mechanism for controlling cognitive activity operating in different domains (attention, memory, language, etc.) both automatically and consciously, thus allowing the inhibition of mental processes and their contents [12, 13].

For cognitive inhibition over memory, some authors have proposed three processes: access processes—controlling the information entering WM—, elimination processes—controlling information removal from WM—, and restriction processes—preventing non-relevant information from entering WM—[14]. If the control of cognitive inhibition on memory (CMI) varies the accessibility of memory traces and their future retrieval, then the failure of CMI processes may lead to the forgetting of relevant information. Optimal operation of CMI promotes maintaining the information to be remembered. Research relating information maintenance in WM to the capacity for cognitive inhibition has found that people with a better executive control capacity perform much better on WM tasks [15]. Similarly, other authors found that a higher maintenance load produced a higher level of interference and an increased demand for executive control [4]. Thus, optimal performance of cognitive inhibition processes is essential for resolving interference from information interrupting the execution of the current task.

This ability to resolve interference is altered in some populations. Several studies have found less efficacious interference resolution in older people [11, 16–18]. These results have been converged with those of neuroimaging studies [19].

Age-related decreases in CMI ability have generated interest in early ageing populations, such as individuals with DS [20–23]. People with DS have more difficulties on CMI tasks than people with ND [24, 25]. In a recent meta-analysis, some weaknesses of the few studies on CMI in people with IDs were detected, including disparities in empirical paradigms, the use of different tasks, very different age ranges, and few tasks in each range [26]. After analysing these weaknesses, some authors conducted a study on CMI processes in adults with DS [27] using a proven experimental paradigm of natural ageing processes in individuals with ND [28, 29]. They applied CMI tasks with low maintenance loads (a single stimulus per trial) using food images as stimuli. In individuals with ND, interference did not decrease performance, but interference affected CMI performance in the DS group.

The results corroborated difficulties in interference resolution in WM tasks in the DS group and found no differences according to the participants' ages. These results, using a low-load task, suggest that the maintenance load is a relevant variable for studying CMI in people with DS.

In addition to the maintenance load variable, another variable that may affect the resolution of interference is the type of stimulus because social and non-social stimuli are processed differently. The results of studies of individuals with ND have demonstrated holistic processing when the stimuli were faces, which differs from object processing (for a meta-analysis, see [30]). Several studies have analysed the influence of social versus non-social materials in interference processes. Some authors found that individuals with ND had more difficulties with CMI resolution when the stimuli were faces than when they were objects [31]. However, other authors found similar difficulties in this population for both objects and faces when the same stimulus was used in the encoding and maintenance phases [32].

All of the studies conducted thus far on CMI in DS have used only non-social stimuli (isolated letters, words, or pictures of everyday objects). Research studying memory processes, but not CMI processes, in people with DS has produced contradictory findings. Some authors did not find differences, while others found worse memory performance for stimuli consisting of both objects and faces compared to control groups, with better results obtained in both groups when recalling faces rather than objects [33]. Several studies have analysed face recall in people with DS. Systematically, difficulties in recalling faces were noted in the DS group compared to the ND group [34–36]. Problems with face processing seem to depend on the aetiology of ID. A broad consensus indicates that these difficulties are observed in people with DS and in those with unspecified ID, but no difficulties have been observed in people with other conditions, such as William Syndrome (WS) [37–41]. The influence of the material (social/non-social) on CMI in people with DS is an open question.

With respect to the populations included in this study, on the one hand, people with DS were selected because this syndrome is considered the most prevalent cause of ID [42], with a known genetic diagnosis and a relatively well-defined neuropsychological profile [43–45]. On the other hand, considering the results of previous studies where the pattern of findings for CMI differed between groups with ND and groups with DS [24, 25], we also included a group with ND to establish whether differences emerge between groups when including the variables maintenance load and stimulus type. In addition, knowing the magnitude of the effect of the distance between both groups on the resolution of interference is relevant from both the theoretical and clinical perspectives.

In a complementary manner, given that CMI is sensitive to the decline associated with age in the population with ND [16–18] and considering that some people with DS have shown premature cognitive deterioration associated with age [23], we analysed the functioning of CMI in adults with DS in relation to age.

The importance of our study lies in the originality of assessing CMI processing in people with DS by incorporating in the same study variables related to the maintenance load using non-social stimuli and also using social stimuli for the first time. Thus, the main goals of this study are as follows:

1. To determine the effect of maintenance load levels (high versus low) on interference resolution in WM tasks in adults with DS;
2. To determine the effect of the stimuli (non-social versus social) on interference resolution in WM tasks in adults with DS; and
3. To determine whether the patterns of results between the group of people with ND and the group of people with DS differ. The magnitude of the effect size between the groups was also determined.

The hypothesis related to the goal of the maintenance load is that lower performance is expected in the interference condition (IC) generated by a concurrent activity while performing a high-load WM task (versus a low-load WM task). The exploratory nature of including the variable social/non-social stimuli does not allow us to formulate a hypothesis related to this goal. The hypothesis related to the third goal is that a differential pattern of CMI is expected between the groups for both variables (the maintenance load and stimulus type).

The secondary objective is to establish whether any relationship exists between CMI and age in adults with DS as observed in people with ND given the premature cognitive ageing noted in some people with DS.

## Materials and methods

### Participants

The sample consisted of 36 individuals with DS (mean age = 33.44 years, standard deviation = 7.54 years; range 20–48 years; 18 men and 18 women) and 36 individuals with ND (mean age = 33.55 years, standard deviation = 7.52 years; range 21–47 years; 18 men and 18 women).

All of the individuals in the DS group were paired one to one with participants in the ND group based on hand dominance, sex, and chronological age (interval ± six months). The participants in the DS group attended occupational centres in the Community of Madrid (Spain).

The inclusion criteria were as follows: I) age older than 18 years; II) intellectual quotient ≥ 35; III) a sufficient attention and auditory comprehension capacity to understand the instructions for the neuropsychological tests and experimental tasks (assessed with the CAMCOG-DS language comprehension section); IV) no hearing loss greater than 30 decibels; V) no chronic neurological disease (e.g., epilepsy or dementia); and VI) no current treatment with first-generation antipsychotics.

This research was approved by the ethics committee of the corresponding university. Similarly, the participants and their family members signed an informed consent form before assessment.

### Neuropsychological assessment

To examine the cognitive capacity of each participant, an individual neuropsychological assessment was performed in both groups with the following tests: the Geriatric Depression Scale (Yesavage) [46]; the Subjective Memory Complaints Questionnaire (SMCQ) [47]; the Functional Assessment Questionnaire (FAQ) [48]; the Global Deterioration Scale (GDS) [49]; the Go/No-go test [50]; and the Working memory visuospatial sketchpad test of the Wechsler Memory Scale—Third Edition (WMS-III) [51]. The last test has been classically used in populations with DS to evaluate the visuospatial sketchpad of the WM [52–55] and is the most frequently used test in the literature to evaluate this component [56]. For this test, the motor requirement of pointing behaviour was guaranteed considering the fine motor skills of the participants in the sample. Within the DS group, the following tests were also applied: the Wechsler Adult Intelligence Scale—Fourth Edition (WAIS-IV) [57] and the Cambridge Examination for Mental Disorders of Older People with Down's Syndrome and Others with ID (CAMDEX-DS) [58]. In the ND group, the Revised Cambridge Examination for Mental Disorders of the Elderly (CAMDEX-R) [59] was administered. The WAIS-IV was not administered in the ND group because the usual criterion of normotypical intelligence was assumed in these participants who were selected individually and met the inclusion and exclusion criteria. Additionally, despite possible individual differences, considering the sample size, intellectual performance in the middle range is expected.

Neuropsychological assessment was performed in the DS group in three sessions of approximately 50 minutes each. Subsequently, the individuals’ families completed the FAQ and GDS questionnaire. The people with ND completed the neuropsychological battery in a session of approximately 75 minutes and later completed the FAQ and GDS questionnaire.

The neuropsychological battery was implemented with two objectives: to detect the presence of cognitive impairment in participants and to determine whether better resolution of interference is related to better results in the tests commonly used to measure different cognitive processes.

### Stimuli and tasks

All of the participants performed the experimental session after a neuropsychological assessment lasting 60 minutes. To assess the operation of CMI processes, a visual recognition paradigm was used [28]. The experimental session consisted of two groups of tasks in which only the stimulus (food or faces) changed. Both tasks consisted of 80 trials distributed in four blocks of 6 minutes each (20 trials per block) separated by a 2-minute interval. The task consisted of three phases: encoding, maintenance, and retrieval (recognition). The stimuli were presented using E-Prime software, version 2.0 (Psychology Software Tools, Pennsylvania, USA) [60]. A 30-minute break was scheduled between the tasks.

For the food task, 200 colour images were selected from the FoodCast research image database [61], Food-pics [62], and Utrecht standardised food images [63]. Images were shown cut out on a white background, and in each trial, the stimuli came from the same database. The stimuli were selected through an inter-judge task in which foods had to be named. Stimuli with 100% inter-judge agreement were selected.

In the face-recognition task, 200 colour images of non-familiar, neutral faces were selected. The face images were cut out in an oval shape and set on a white background without ears or hair to prevent specific details from guiding the memory. The ages and genders of the face images were controlled, and fifty faces were selected from each group: young women, older women, young men, and older men. The stimuli were selected through an inter-judge task, and only stimuli with 100% inter-judge agreement were selected. For the inter-judge task, the judges informed whether an image depicted a man or woman and the corresponding age group.

A Hewlett-Packard personal computer was used to present the experimental task. Stimuli were projected at a distance of 50 cm and focused on the fovea. Throughout the experimental task, feedback was not offered to the participants regarding correct or incorrect responses.

Before beginning the training phase, all participants completed the instruction-comprehension phase. The evaluator ensured that all individuals in the DS and ND groups understood the concepts "inside the house", “salty”/“sweet", and “man”/“woman”, each of which was evaluated using 10 images. If a participant provided 100% correct answers, then the instructions for the task were explained. Once the evaluator ascertained that the instructions were understood, the training phase was started, followed by the experimental phase. The experimental task consisted of four conditions: two low-load conditions (no interference and interference) and two high-load conditions (no interference and interference). For all conditions, the trials of the encoding phase began with a black-and-white image of an eye to denote commencement and focus the participant's attention. Then, in the case of a low-load condition, the stimulus (a food image or a face image according to the task) appeared inside a picture of a house for 1500 ms. In the case of a high-load condition, after this first stimulus was presented, another stimulus appeared inside the picture of a house for 1500 ms. The participants were instructed to memorise any images inside the house. During the maintenance interval, the participant first saw a blank screen with a cross in the centre (fixation point) for 1000 ms. Then, two situations could occur: in the no-interference condition (NIC), the blank screen with the cross remained for another 1500 ms, and in the IC, another stimulus that was not framed inside a house and was different from the previous one appeared for 1500 ms. In addition, to ensure interference (in this case, an interruption in processing), the participants were asked a question. In the food task, in half of the trials, the participants were asked, "Is it salty?", and in the other half, "Is it sweet?" In the face task, in half of the trials, the participants were asked, "Is it a woman?", and in the other half, "Is it a man?" Then, in both conditions, the screen was kept blank for 4000 ms with a cross in its centre to focus attention.

The recognition phase was identical for all of the conditions and tasks. First, for 1000 ms, an image of a house was presented with two question marks on the sides. Then, for 1500 ms, an image (food or a face depending on the task) was presented inside the house between question marks. At the same time, the participant listened to the phrase "Was it in the house before?" In half of the presentations, the image for the recognition phase was the same as that for the encoding phase, and in the other half, the image was different. A randomised distribution of the trials was applied. Under high-load conditions, when asked "Was it in the house before?", the participant had to answer yes if the stimulus that he/she saw was one of the two stimuli that he/she had seen in the encoding phase. Under all conditions, the participants used headphones to listen to pre-recorded questions. Subsequently, the food or face image disappeared, but the house image with the question marks remained until the participant gave an answer (affirmative/negative). The participant responded using two buttons: a green button for affirmative responses and a red button for negative responses. The participants placed the buttons such that the "yes" (green button) option was next to their dominant hands.

**Fig 1:**
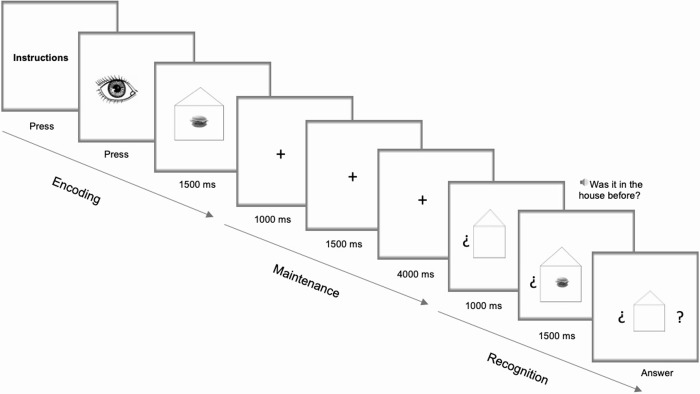
Low-load condition without interference: With objects as stimuli.

**Fig 2:**
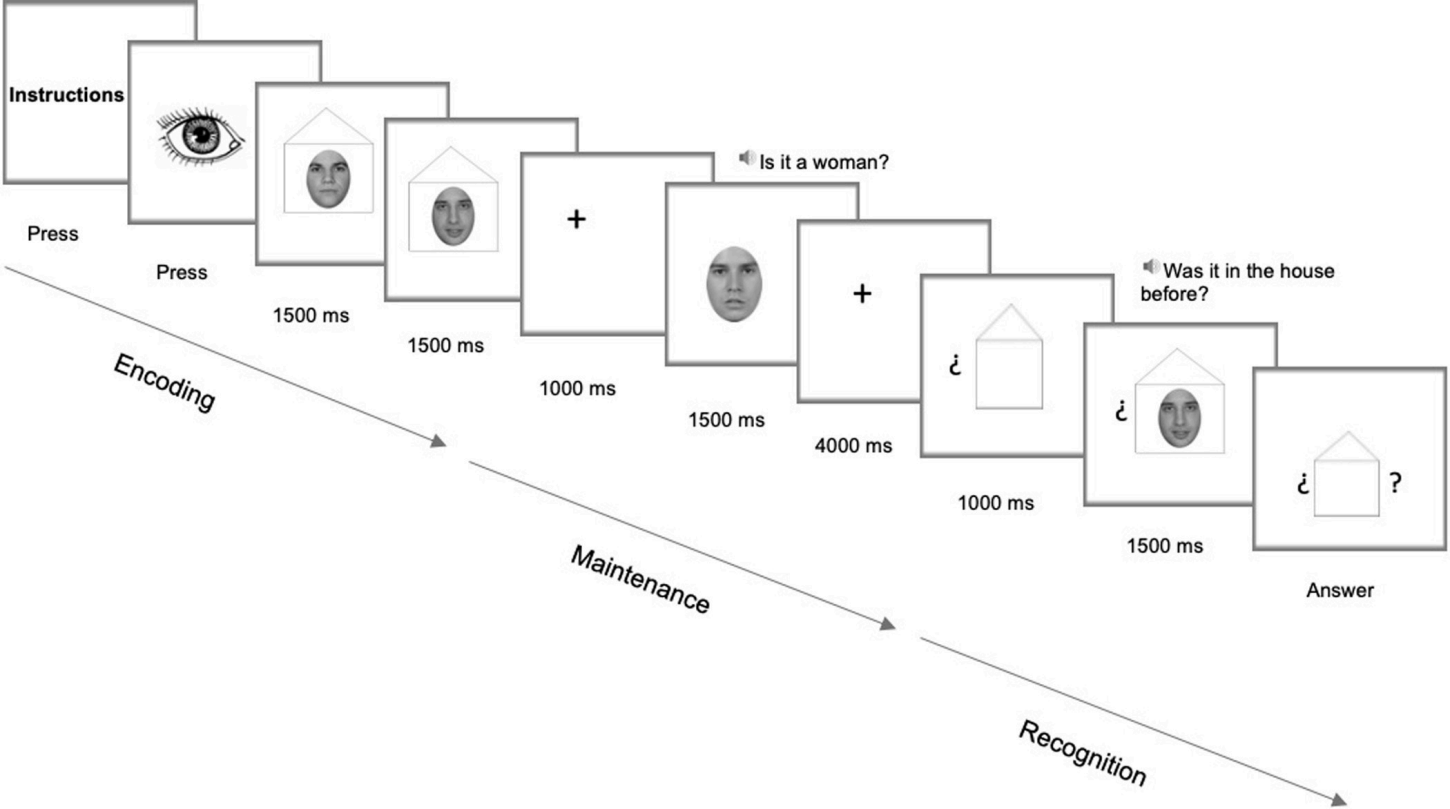
High-load condition with interference: With faces as stimuli.

When the participant gave the recognition response, a blank screen (250 ms) appeared, followed by a screen with a drawing of an eye indicating the beginning of the next test (see examples of low and high load in Figs 1 and 2, respectively).

### Design

Four independent variables were manipulated: one between-participant variable (population) and three within-participant variables (load, material, and interference). The population variable had two levels: individuals with DS and individuals with ND. The load variable had two levels: low (single-item maintenance) and high (two-item maintenance). The stimulus variable had two levels: food pictures and face pictures. The interference variable had two levels: no interference (no new information was presented during the maintenance phase) and interrupting interference (a concurrent task was performed during the retention interval). The factorial design was 2 x 2 x 2 x 2. The dependent variable was the number of correct responses.

### Procedures

Experimental tasks were randomised such that half of the sample first performed the task with food stimuli and the other half first performed the task with face stimuli. The experimental conditions were presented first under a low load and then under a high load and always in the same order: first without interference and then with interference interruption. Thus, the baseline operation of the groups could be established under the different conditions without the presence of interference.

### Statistical analysis

SPSS software version 20 was used (IBM, New York, USA) [64]. The assumption of normality was assumed, and subsequently, whether the assumption of homoscedasticity of variances was fulfilled was evaluated. In addition, the effect size was calculated; in analyses of more than two groups, the partial eta-squared was used ($\eta_{p}^{2}$). An effect was interpreted to be small when $\eta_{p}^{2}$ = .01, medium when $\eta_{p}^{2}$ = .06, and large when $\eta_{p}^{2}$ = .14. For analyses of two groups, Cohen's *d* was used, and the effect was interpreted as a small when *d* = 0.2, medium when *d* = 0.5, and large when *d* = 0.8. Regarding the direction according to the analysis, a positive sign for both statistical parameters and effect sizes indicated more memory in the NIC, more memory in the ND group, more memory in the condition with objects as stimuli, or more memory under low-load conditions.

The presence of cognitive impairment in participants in both groups was analysed. The detection criteria were a lower score than the cut-off point for the CAMDEX, a GDS score > 2, the presence of subjective memory complaints, and an SMCQ questionnaire score > 2. No participants met the criteria established for cognitive impairment; therefore, all participants were part of the analysed sample. No missing values were noted in either the neuropsychological assessment or the experimental task.

## Results

### Neuropsychological assessment

Significant differences were identified, with better performance observed in the ND group on the CAMDEX test, the visuospatial sketchpad test of the WMS-III, the Go/No-go test in terms of the number of omission errors, the Yesavage test, and the FAQ (Table 1).

**Table 1:** Neuropsychological assessment by group: Mean (standard deviation).

Assessment	DS	ND
Cognitive impairment detection		
Camdex	82.5 (7.28)	99.03 (3.44)
GDS	1 (0)	1 (0)
QSM	0.47 (0.77)	0.25 (0.5)
Intelligence		
WAIS	41.75 (3.78)	-
Working memory system		
WMS-Visuospatial sketchpad	6.19 (2.45)	14.78 (3.42)
Executive function		
Go/No-Go		
Omission errors	39.06 (39.58)	0.72 (1.39)
Commission errors	7.5 (8.29)	5.06 (4.46)
Emotional problems		
Yesavage	1.56 (1.05)	0.92 (1.36)
Functional activity		
FAQ	14.58 (6.67)	0 (0)

DS, Down syndrome; ND, neuro-typical development; -, no data recollection.

These results show that people with DS presented more difficulties in general cognitive performance, in the amplitude of the visuospatial sketchpad of the WM, in processing speed (in relation to omission errors in the Go /No-go test), in the resolution of emotional problems, and in the execution of functional activities of daily life.

### Load effects

To determine the effects of the maintenance load (low and high), repeated-measures analysis of variance (ANOVA) was performed with the variables population, load, and interference level without considering the material. The following findings were identified: I) the main effect of the population variable: *F* (1.70) = 353.42, *p* < .001, $\eta_{p}^{2}$ = .84, indicating that individuals with DS (mean = 12.58, standard deviation (*SD*) = 1.26) showed lower performance than individuals with ND (mean = 17.55, *SD* = 0.98); II) the main effect of the load variable: *F* (1.70) = 51.98, *p* < .001, $\eta_{p}^{2}$ = .43, indicating that recall was better under low-load conditions (mean = 15.62, *SD* = 2.72) than that under high-load conditions (mean = 14.51, *SD* = 2.91); III) the main effect of the interference variable: *F* (1.70) = 29.11, *p* < .001, $\eta_{p}^{2}$ = .29, reflecting lower recall under conditions requiring interference (interruption) resolution during the maintenance phase (mean = 14.63, *SD* = 3) than that under NICs (mean = 15.5, *SD* = 2.66); IV) the effect of the interaction between population and interference: *F* (1.70) = 7.39, *p* = .008, $\eta_{p}^{2}$ = .1—; and V) the effect of the three-way interaction among population, load, and interference: *F* (1.70) = 32.40, *p* < .001, $\eta_{p}^{2}$ = .32. The interaction between population and load was not statistically significant: *F* (1.70) = 0.82, *p* = .368, $\eta_{p}^{2}$ = .01, power = .15. The interaction between load and interference was not statistically significant: *F* (1.70) = 0.45, *p* = .503, $\eta_{p}^{2}$ = .01. Overall, we observed lower performance in the DS group, better performance under low loads, and worse performance in the presence of interference.

In the DS group, significant differences were observed between low-load NICs and low-load ICs: t(35) = 5.15, *p* < .001, *d* = 0.86. No significant differences were found between high-load NICs and ICs: t(35) = 1.58, p = .123, *d* = 0.26, power = .36. In the DS group, we found that under a low load, the individuals were influenced by interference, while under a high load, they were not influenced by interference. In the ND group, under low-load NICs and ICs, no statistically significant differences were found: t(35) = - 1.56, *p* = .127, *d* = - 0.26, power = .36. In contrast, the differences between high-load NICs and ICs were significant: t(35) = 4.3, *p* < .001, *d* = 0.72. In the ND group, the pattern of results was the opposite of that of the DS group, and interference influenced the individuals only under the condition of a high maintenance load.

When comparing performance in the DS group, significant differences were observed in the recognition task under low- and high-load NICs: t(35) = 7.47, *p* < .001, *d* = 1.24. Recognition was lower under high-load conditions than that under low-load conditions. However, no statistically significant differences were observed between low-load ICs and high-load ICs: t(35) = 1.06, *p* = .298, *d* = 0.18, power = .2. In this group, in the absence of interference, performance worsened when we asked the individuals to retain more information in WM. However, once we introduced interference, performance under the low-load condition was affected. In the ND group, no differences were observed between the low-load NICs and high-load NICs: t(35) = 1.22, *p* = .23, *d* = 0.2, power = .22. In contrast, significant differences were observed between low-load ICs and high-load ICs: t(35) = 6.66, *p* < .001, *d* = 1.11. The pattern of results in the ND group was the exact opposite of that the DS group, and the load level influenced performance only in the presence of interference. In the DS group, this relationship appeared under a low maintenance load or in conditions without interference. However, in the ND group, this relationship occurred under a high maintenance load or in conditions with interference. Thus, the interference variable was found to be related to the variable load as a function of the group.

A between-group analysis was performed, and under the low-load NIC, individuals with ND recognised more information than those in the DS group: t(51) = 10.42, *p* < .001, *d* = 2.46. This same pattern was found under low-load ICs: t(54) = 15.81, *p* < .001, *d* = 3.79, indicating that the DS group exhibited lower performance than the ND group. Additionally, under high-load NICs, individuals in the DS group remembered fewer words than those in the ND group: t(70) = 13.55, *p* < .001, *d* = 3.25. Under high-load ICs, individuals in the ND group showed better performance in delayed image recognition than individuals in the DS group: t(70) = 13.55, *p* < .001, *d* = 3.25 (Tables 2–4). In the intergroup analysis, we found that in all conditions, the ND group had greater recall than the DS group.

**Table 2:** Experimental task by load: Mean (standard deviation).

Experimental condition	N = 72	DS	ND
Low load	15.62 (2.72)	13.2 (1.47)	18.03 (0.9)
High load	14.51 (2.9)	11.96 (1.41)	17.07 (1.33)

DS, Down syndrome; ND, neuro-typical development.

**Table 3:** Experimental task by interference: Mean (standard deviation).

Experimental condition	N = 72	DS	ND
No interference	15.51 (2.66)	13.24 (1.64)	17.77 (1.07)
With interference	14.63 (3)	11.92 (1.39)	17.33 (1.08)

DS, Down syndrome; ND, neuro-typical development.

**Table 4:** Experimental task by load and interference: Mean (standard deviation).

Experimental condition	N = 72	DS	ND
Low load: No interference	16.10 (2.34)	14.29 (1.87)	17.92 (0.92)
Low load: With interference	15.13 (3.44)	12.11 (2.01)	18.15 (1.09)
High load: No interference	14.91 (3.21)	12.19 (1.82)	17.63 (1.57)
High load: With interference	14.12 (2.84)	11.72 (1.5)	16.51 (1.5)

DS, Down syndrome; ND, neuro-typical development.

### Material effects

The differential effects of the material (non-social versus social) on performance under different experimental conditions were analysed. In the DS group, differences were found in low-load NICs depending on the stimulus: t(35) = 2.18, *p* = .036, *d* = 0.36. Food stimuli were better remembered than faces. Under low-load ICs, the differences were also significant: t(35) = 4.13, *p* < .001, *d* = 0.69. Memory for faces was lower than that for food. Regarding high-load conditions, memory was significantly better in NICs with food than that with faces: t(35) = 2.95, *p* = .006, *d* = 0.49. In high-load ICs, the differences between the conditions were significant: t(35) = 2.59, *p* = .014, *d* = 0.43. Specifically, food stimuli were better remembered than faces. In the DS group, a homogeneous pattern was observed. Specifically, in all conditions, the individuals with DS remembered food as a stimulus better than faces.

In the ND group, the differences between faces and food were not significant under low-load NICs: t(35) = 1.21, *p* = .235, *d* = 0.2, power = .22. No significant differences between food and faces were found in low-load ICs: t(35) = - 0.09, *p* = .928, *d* = - 0.02, power = .17. In high-load NICs, no significant differences were found between the conditions with faces and food as stimuli: t(35) = - 1.54, *p* = .134, *d* = - 0.26, power = .36. The same outcome occurred under high-load ICs, with no significant differences between food and face stimuli: t(35) = 1.28, *p* = .208, *d* = 0.21, power = .26. In all conditions, the ND group had similar results when faces and food were presented as stimuli. Subsequently, the effects of the levels of the load and interference variables for each stimulus were analysed (Tables 5 and 6).

**Table 5:** Experimental task by material: Mean (standard deviation).

Down syndrome		
Experimental condition	Food	Faces
Low load: No interference	14.86 (2.38)	13.72 (2.50)
Low load: With interference	12.97 (2.24)	11.25 (2.50)
High load: No interference	13.11 (2.28)	11.28 (2.89)
High load: With interference	12.22 (2.07)	11.22 (1.71)

**Table 6:** Experimental task by material: Mean (standard deviation).

Neurotypical development		
Experimental condition	Food	Faces
Low load: No interference	18.08 (1.05)	17.75 (1.40)
Low load: With interference	18.14 (1.13)	18.17 (1.68)
High load: No interference	17.36 (2.09)	17.89 (1.65)
High load: With interference	16.78 (1.73)	16.25 (2.13)

#### Task with food stimuli

Repeated-measures ANOVA was performed with the variables population, load, and interference level. The findings were as follows: I) the main effect of the population variable: *F*(1.70) = 228.36, *p* < .001, $\eta_{p}^{2}$ = .77, indicating that recognition by individuals with DS (Mean = 13.29, *SD* = 1.35) was lower than that of individuals with ND (mean = 17.59, *SD* = 1.04); II) the main effect of the load variable: *F*(1.70) = 24.88, *p* < .001, $\eta_{p}^{2}$ = .26, indicating that the participants recognised more information under low-load conditions (mean = 16.01, *SD* = 2.53) than under high-load conditions (mean = 14.87, *SD* = 2.78); and III) the main effect of the interference variable: *F*(1.70) = 20.82, *p* < .001, $\eta_{p}^{2}$ = .23, indicating that recognition of previously presented foods decreased when the participant had to solve a task during the maintenance phase (mean = 15.03, *SD* = 2.85) compared to NICs (mean = 15.85, *SD* = 2.34). The interaction between population and interference was significant: *F*(1.70) = 9.65, *p* = .003, $\eta_{p}^{2}$ = .12. In contrast, no effects of the interaction between interference and load were observed: *F*(1.70) = 0.2, *p* = .656, $\eta_{p}^{2}$ .00, power = .07. No effects of the interaction between population and load were found: *F*(1.70) = 0.21, *p* = .652, $\eta_{p}^{2}$ = .00, power = .07. Finally, a significant effect was found for the three-way interaction among population, load, and interference: *F*(1.70) = 4.12, *p* = .046, $\eta_{p}^{2}$ = .06. Overall, we observed lower performance in the DS group, better performance under a low load, and worse performance in the presence of interference.

In the DS group, under low-load NICs or high-load NICs, significant differences were observed compared to their respective ICs (interruption): low load, t(35) = 3.82, *p* = .001, *d* = 0.64; high load, t(35) = 2.12, *p* = .041, *d* = 0.35. For food stimuli, in the DS group, the presence of interference was related to lower performance regardless of the load. In the ND group, under low-load NICs or high-load NICs, no significant differences were found compared to their respective ICs (interruption): low load, t(35) = - 0.26, *p* = .797, *d* = - 0.05, power = .17; high load, t(35) = 1.67, *p* = .105, *d* = 0.28, power = .4. For food stimuli, the ND group was not affected by interference in any of the load conditions.

When comparing performance in the DS group, significant differences were observed in the recognition task under low-load and high-load NICs: t(35) = 2.98, *p* = .005, *d* = 0.5. Recognition of the food images was lower under high-load NICs than that under low-load NICs. In contrast, no statistically significant differences were observed between the low-load ICs and high-load ICs: t(35) = 1.69, *p* = .1, *d* = 0.28. In the DS group, in the absence of interference, the individuals were influenced by the load. However, in the presence of interference, performance was similar across all loading conditions. In the ND group, differences were observed under low-load NICs and high-load NICs: t(35) = 2.02, *p* = .05, *d* = 0.33. Additionally, significant differences were observed between low-load ICs and high-load ICs: t(35) = 4.87, *p* < .001, *d* = 0.81. This group was influenced by the maintenance load regardless of whether interference was present.

A between-group analysis was performed, and under low-load NICs, individuals with ND recognised more information than those in the DS group: t(48) = 7.43, *p* < .001, *d* = 1.75. This same pattern was found under low-load ICs: t(52) = 12.39, *p* < .001, *d* = 2.97, indicating that the DS group had lower performance than the ND group. Similarly, under high-load NICs, individuals in the DS group remembered fewer words than those in the ND group: t (70) = 8.26, *p* < .001, *d* = 1.98. Under high-load ICs, individuals in the ND group showed better performance in delayed recognition of food images than individuals in the DS group: t(70) = 10.14, *p* < .001, *d* = 2.43. Based on the between-group analysis, the ND group had better results than the DS group across all conditions.

#### Task with face stimuli

The effects of interference and the maintenance load on WM task resolution with face images as stimuli were analysed. For this purpose, repeated-measures ANOVA was performed with the variables population, load, and interference level. The following results were found: I) the main effect of the population variable: *F* (1.70) = 309.41, *p* < .001, $\eta_{p}^{2}$ = .82, indicating that individuals with DS (mean = 11.87, *SD* = 1.52) had lower performance than individuals with ND (mean = 17.51, *SD* = 1.19); II) the main effect of the load variable: *F* (1.70) = 29.28, *p* < .001, $\eta_{p}^{2}$ = .30, indicating that the participants recalled more information under low-load conditions (mean = 15.22, *SD* = 3.16) than under high-load conditions (mean = 14.16, *SD* = 3.35); and III) the main effect of the interference variable: *F* (1.70) = 16, *p* < .001, $\eta_{p}^{2}$ = .19, indicating that recall decreased under conditions in which the participant had to resolve the interference caused by an interruption during the maintenance phase (mean = 14.22, *SD* = 3.37) compared to NICs (mean = 15.16, *SD* = 3.24). However, the interaction between interference and load was not statistically significant: *F*(1.70) = 0.16, *p* = .695, $\eta_{p}^{2}$ = .00, power = .07. Additionally, the interaction between population and interference was not statistically significant: *F* (1.70) = 1.94, *p* = .168, $\eta_{p}^{2}$ = .03, power = .28. The interaction between population and load was not statistically significant: *F* (1.70) = 0.78, *p* = .38, $\eta_{p}^{2}$ = .01, power = .14. Finally, a statistically significant effect was found for the three-way interaction among population, load, and interference: *F* (1.70) = 23.76, *p* < .001, $\eta_{p}^{2}$ = .25. Overall, lower performance was observed in the DS group, better performance was observed under a low load, and worse performance was observed in the presence of interference.

In individuals with DS, significant differences were observed under low-load conditions between NICs and ICs (interruption): t(35) = 4.2, *p* < .001, *d* = 0.7. However, under high-load conditions, execution under NICs and ICs was similar. Therefore, the presence of interference did not affect information recall under high-load conditions: t(35) = 0.1, *p* = .919, *d* = 0.02, power = .17. In this group, interference was related to a decrease in performance only in the presence of a low maintenance load. In the ND group, no statistically significant differences were observed under low-load conditions between NICs and ICs: t(35) = - 1.57, *p* = .125, *d* = - 0.26, power = .36. In contrast, in this population, under high-load conditions, the presence of interference in the maintenance phase caused a significant decrease in information recall compared to NICs: t(35) = 4.22, *p* < .001, *d* = 0.7. In the ND group, interference was related to a decrease in performance only in the presence of a high maintenance load.

For individuals with DS, NICs were compared under low-load and high-load conditions, which revealed statistically significant differences: t(35) = 4.95, *p* < .001, *d* = 0.82. Specifically, lower recall was observed under high-load conditions than under low-load conditions. In contrast, no statistically significant differences were observed between low-load ICs and high-load ICs: t(35) = 0.06, *p* = .96, *d* = 0.01. In this group, the load influenced performance only under conditions with no interference. For individuals with ND, no differences were observed between low-load NICs and high-load NICs: t(35) = - 0.49, *p* = .63, *d* = - 0.08. However, significant differences were observed between low-load ICs and high-load ICs: t(35) = 4.69, *p* < .001, *d* = 0.78. In this group, the load influenced performance only in conditions with interference.

A between-group analysis was performed, and under low-load NICs, individuals with ND recalled significantly more information than those in the DS group: t(55) = 8.43, *p* < .001, *d* = 1.99. The same pattern was found under low-load ICs: t(61) = 13.77, *p* < .001, *d* = 3.25, indicating that the DS group had lower performance than the ND group. Similarly, under high-load NICs, individuals in the DS group remembered fewer words than those in the ND group: t(56) = 11.9, *p* < .001, *d* = 2.81. Finally, in high-load ICs, individuals in the ND group had more effectively recalled delayed information than individuals in the DS group: t(67) = 11.05, *p* < .001, *d* = 2.6. Based on the between-group analysis, the ND group had better results than the DS group across all conditions.

#### Complementary analyses

Considering the early age-related impairment observed in IDs, specifically in DS and WS [23, 65], the relationship between age and CMI was studied. Because such impairment is considered to emerge at 35 years of age [66, 67], we established two groups: younger (up to 35 years) and older (over 35 years).

In the task using objects as stimuli, interference affected younger and older individuals in the DS group equally under low-load conditions (t(34) = 0.11, *p* = .912, *d* = 0.3, power = .17) and high-load conditions (t(34) = 1.2, *p* = .24, *d* = 0.4, power = .22). Likewise, interference similarly affected younger and older individuals with ND under low-load conditions (t(34) = 0.26, *p* = .8, *d* = 0.09, power = .17) and high-load conditions (t(27) = 0.39, *p* = .69, *d* = 0.13, power = .17).

In the task using faces as stimuli, interference equally affected younger and older individuals in the DS group under low-load conditions (t(34) = 0.89, *p* = .378, *d* = 0.3, power = .56) and high-load conditions (t(27) = 0.30, *p* = .763, *d* = 0.1, power = .26). Likewise, interference similarly affected younger and older individuals with ND under low-load conditions (t(34) = 0.52, *p* = .608, *d* = 0.18, power = .29) and high-load conditions (t(34) = 0.78, *p* = .44, *d* = 0.26, power = .48).

Because a relationship between age subgroups and CMI performance was not observed, the suitability of the selected cut-off point was analysed. For this purpose, two hierarchical conglomerations between age and CMI resolution were performed within each group. The results did not justify changing the cut-off point for age.

Additionally, other complementary analyses were performed, and the correlations between the neuropsychological tests and the experimental task results were calculated. The Pearson correlation was calculated between the average number of correct responses in the experimental conditions and performance in the neuropsychological tests. In the DS group, higher overall performance on the CAMDEX corresponded to better scores in high-load conditions with interference, and a higher intelligence quotient was correlated with better results for the resolution of interference. In the ND group, the optimal resolution of interference was related to better results for visuospatial WM.

## Discussion

This study analysed interference resolution in adults with DS and adults with ND using a delayed visual recognition task. Although we assessed participants with a broad age range (20 to 48 years old), no statistically significant differences were found for age.

Regarding the analysis of the relationships between the clinical and experimental data, to the authors’ knowledge, this study is the first to examine CMI processes in people with DS and identify significant correlations. We found that better resolution of interference was correlated with higher scores on the CAMDEX and the WAIS-IV.

In all of the global between-group comparisons, the CMI results were more unfavourable in the DS group than those in the ND group. Regarding the experimental paradigm (unpublished in people with DS), the overall results in both groups showed a general interference effect, with better performance under NICs. The task used was adequate for the proposed goals and the samples evaluated since we did not find a ceiling or floor effect under any condition.

Overall, the DS group had more difficulties in CMI resolution than the ND group (with both food and face stimuli). In addition, the effect sizes were interpreted as large and were consistent with those of previous studies (for a meta-analysis, see [26]).

Regarding the first goal of the study (to study the effects of maintenance load levels on CMI tasks in people with DS and ND), an expected load effect was observed globally in both groups, with worse resolution under high-load conditions. The results in the DS group were worse than those in the ND group under both low-load and high-load conditions. Significant differences were found under high-load NICs in the DS group. However, in the ND group, no high-load effect was found, and the increase in load did not hinder execution. No differential effects related to the load under ICs were found in the group with DS, and execution seemed to be affected even under low-load conditions. However, in the ND group, significant differences were found with the increase in load, with an increase from one stimulus to two stimuli affecting CMI execution.

Regarding the second goal of the study (analysing the influence of social/non-social stimuli), the DS group had worse results overall than the ND group regardless of the stimulus. When comparing both types of stimuli, no significant differences were found in the ND group. These results are consistent with those of previous studies in which execution in the ND group was similar regardless of the material [32, 68]. However, in individuals with DS, better performance was found with non-social stimuli (food) than with social stimuli (faces) regardless of the load and interference levels.

By analysing the variables population, load, and interference together, a significant three-way interaction was observed when the stimuli were food and when they were faces. Considering only the results when the stimuli were food, in the DS group, under all of the load conditions, worse results were found with interference; introducing interference always negatively affected individuals in the DS group. The increase in load only produced effects when no interference was present, but under ICs no negative effect of an increased load was noted. Accordingly, we deduce that interference affects CMI in this group even under low-load conditions. However, in the ND group, no significant differences were observed when interference was introduced under low-load or high-load conditions. Increasing the load affected this group under both ICs and NICs. However, under NICs, the magnitude of the effect size was small, and under ICs, the magnitude of the effect size was large. Comparisons between the two groups showed significant differences in the two interference conditions and in the two load levels, with all of the effect sizes being large. These results indicate an important effect on CMI in the DS group under different difficulty conditions using food as stimuli.

When the stimuli were faces, the interference effect was observed only under low-load conditions. Under high-load conditions, performance was affected even when no interference was present. In general, the increase in load when the stimuli were faces led to decreases in the resolution capacity of CMI. Specifically, when no interference was present, load affected performance. In contrast, when interference was present, scores were low under both low-load and high-load conditions. We understand that interference affects processing so negatively that a higher load does not harm processing further. In the ND group, under low-load conditions, introducing interference did not affect the results. However, under high-load conditions, interference affected processing when introduced. These results in young people and young adults with ND confirm the evaluative potential of the design used, and they are consistent with previous results for the effect of load on ICs [2, 69]. In between-group comparisons, significant differences were identified in the two interference conditions and in the two load levels, with all of the effect sizes being large. These results also indicate the important involvement of CMI in the DS group when faces or food were used as stimuli. By comparing the overall results based on the material, better results were observed in the DS group when using food than when using faces. In the ND group, no differences related to the material were found.

These results regarding the material are novel since we have not found previous studies on CMI in individuals with DS in which social and non-social stimuli are compared. We hypothesise that interference resolution, depending on the material, may be related to the aetiology of IDs. In research on face recall in individuals with DS, difficulties have been observed when recalling this material [38, 40, 70]. However, other authors observed that in individuals with WS, face recall in WM tasks was better [71]. Thus, in future research, CMI operations must be studied in people with IDs of different aetiologies to compare the effects with social and non-social stimuli.

## Conclusions

The association of worse CMI operation with ageing and impairment processes has recently shifted the focus of study on early ageing populations, such as individuals with IDs. Since CMI is a partly conscious process, CMI may be the target of neuropsychological interventions similar to cognitive stimulation programmes for CMI developed for the elderly [72]. To plan interventions that enhance CMI in adults with DS, very detailed information about the variables influencing its operation must be acquired to determine the activities that are the most convenient and the materials with which they should be developed. A potential CMI intervention for adults with DS would start from 18 years of age, and programmes designed for people older than forty years should not be applied to this population.

### Limitations of the study

One of the limitations of the study is the homogeneity of the sample. However, DS (or any other developmental condition) is not homogenous because a wide spectrum of variation exists. The evaluated sample represents only one subgroup of DS with sufficient abilities to understand the tasks. Another possible limitation is that no neurophysiological measures were implemented. Such measures will give us information about differential brain activity in different populations during the performance of tasks.

### Clinical implications

Cognitive intervention programmes have been developed for populations with DS, but CMI is infrequently considered in such programmes. CMI is fundamental in all aspects of our lives. The difficulties related to CMI observed in adults with DS demonstrate the need for action plans that simplify simultaneous stimulation in everyday contexts. The results of this study have repercussions, for example, for the design and adjustment of employment positions and training contexts and daily life, among others. Moreover, considering the results, special importance should be assigned to work with social stimuli, at least in groups of individuals with DS, since these stimuli are very important in everyday interactions that we have with various people. These aspects may not be generalisable to all people with different ID conditions. However, individualised assessments and adjustments of intervention programmes are necessary, which will help delineate the evolution of CMI processes in each case.

### Future studies

The next step that we envision is performing magnetoencephalographic (MEG) recordings of the same participants to assess the neurocognitive implications of CMI involvement in WM. Likewise, we will develop a tutorial for an intervention programme favouring CMI strategies. This programme will be developed by professionals in centres for participants with DS. Additionally, the design of future work can focus on longitudinal follow-up studies as well as programmes based on action research on CMI for adults with DS.

### Communication aids for people with DS

The results of this research may lead to active modification of the information environments of adults with DS to facilitate the avoidance of interference in cognitive processing. These suggestions are equally applicable to communication processes: possible interferences and attention allocation can be analysed to provide more support regarding whether information is transmitted only through social stimuli.

## Supporting information

S1 Dataset Experimental data of the study. L, low; H, high; O, object; F, face; NI, no interference; I, interference; Group 1, Down syndrome; Group 2, neuro-typical development; Age 1, young adults; Age 2, old adults.
(XLSX)
